# Protein Determination—Method Matters

**DOI:** 10.3390/foods7010005

**Published:** 2018-01-01

**Authors:** Hanne K. Mæhre, Lars Dalheim, Guro K. Edvinsen, Edel O. Elvevoll, Ida-Johanne Jensen

**Affiliations:** Norwegian College of Fishery Science, Faculty of Biosciences, Fisheries and Economics, UIT The Arctic University of Norway, N-9037 Tromsø, Norway; lars.dalheim@uit.no (L.D.); guro.k.edvinsen@uit.no (G.K.E.); edel.elvevoll@uit.no (E.O.E.); ida-johanne.jensen@uit.no (I.-J.J.)

**Keywords:** proteins, amino acids, analytical methods, extraction methods, Kjeldahl, Bradford, Lowry

## Abstract

The reported protein content of foods depends on the analytical method used for determination, making a direct comparison between studies difficult. The aim of this study was to examine and compare protein analytical methods. Some of these methods require extraction preceding analysis. The efficacy of protein extraction differs depending on food matrices and thus extraction yield was determined. Overall, most analytical methods overestimated the protein contents. The inaccuracies were linked to indirect measurements, i.e., nitrogen determination and subsequent conversion to protein, or interference from other chemical substances. Amino acid analysis is the only protein analysis method where interfering substances do not affect the results. Although there is potential for improvement in regards to the hydrolysis method, we recommend that this method should be the preferred for food protein determination.

## 1. Introduction

Proteins have a major role in the growth and maintenance of the human body and are, along with carbohydrates and lipids, the energy giving nutrients in the diet. In addition, proteins also pose a wide range of other functions in the body, such as enzymatic activity and transport of nutrients and other biochemical compounds across cellular membranes [1]. In order to maintain these important functions, it is essential to provide the body with good quality proteins through diet. Inadequate intake of dietary proteins containing essential amino acids results in increased turnover of muscular proteins, leading to reduced growth and loss of muscle mass. Impaired immunity, as well as reduced hormonal and enzymatic activity may subsequently follow [2]. Being such important constituents of human diet it is crucial to know the protein content in foods and thus it is important to have reliable analytical methods. 

Food protein analysis is not necessarily a straightforward procedure. This is partly due to foods being heterogenic materials, comprised of a range of different nutrients, such as lipids, carbohydrates and a variety of micronutrients. Composition, food structure, or matrix, and interactions between the different nutrients may reduce the accessibility of the proteins leading to underestimation of the protein content. In addition, different methods are based on different analytical principles, determining protein content either directly or indirectly. Direct protein determination is when protein content is calculated based on the analysis of amino acid residues. Indirect protein determination can for instance be inferred following the determination of the nitrogen content, or after chemical reactions with functional groups within the protein. An additional factor that can contribute to inaccuracies in the determination of protein content is protein extraction. Some methods require some degree of protein extraction prior to analysis and thus, extraction yields can affect the results [3]. 

Varieties of different analytical methods have been developed throughout the years. Only a few of these however, are frequently used, and the reason for the choice of method used in many studies is seldom described. This may be due to a variety of factors, for instance tradition (using established analytical procedures in laboratories), lack of analytical infrastructure or high economic costs associated with certain methods. For instance, in a recent review by Angell et al. [4] it was shown that 52% of all studies on the protein content of seaweeds used nitrogen determination with subsequent conversion using a nitrogen-to-protein conversion factor of 6.25. This is in spite of many studies documenting that this factor leads to an overestimation of the protein content in most foods and, in particular, plant foods [5,6,7]. 

All of these variables make performing traditional reviews of studies concerning protein content of foods quite difficult. Thus, the main objective of this study was to document how the determined protein content in several common foods with different matrix compositions varies as a result of the choice of extraction and analytical method. The chosen foods were one lean and one fatty fish (cod and salmon), one crustacean (shrimp), one marine plant (dulse, red seaweed), as well as whole and white wheat flour. The chosen analytical methods were amino acid analysis, Kjeldahl’s method, Bradford’s method and a modified version of the Lowry method. The two extraction solutions were one consisting of a combination of salt and alkaline solutions, and one using a so-called Good’s buffer [8].

## 2. Materials and Methods

### 2.1. Raw Materials

Salmon (*Salmo salar*) loins (*n* = 5) were purchased frozen in local supermarkets in Tromsø (Norway) and kept at −20 °C. Cod (*Gadus morhua*) (*n* = 5) was caught with a fishing rod off the coast of the Lofoten islands (Norway), gutted, put on ice and *post rigor* filleted. Loins were frozen at −20 °C prior to analysis. Batches (100 g) of peeled shrimp (*Pandalus borealis*) (*n* = 5) were obtained frozen from Marealis AS^®^ (Tromsø, Norway) and kept at −20 °C. Prior to analysis, salmon, cod and shrimp samples were thawed and homogenized. White flour (*n* = 5) and whole flour (*n* = 5) of wheat were purchased at local supermarkets. Dehydrated samples of the red seaweed dulse (*Palmaria palmata*) (*n* = 5), harvested at the south coast of Iceland, were purchased from “The Northern Company” (Oslo, Norway). The flour and seaweed samples were kept dark at room temperature until analyses.

### 2.2. Protein Extraction Methods

#### 2.2.1. The Salt/Alkaline Extraction Method

The salt/alkaline extraction was performed as described by Mæhre et al. [9], with minor modifications. Briefly, 0.5 g of raw material was homogenized with 30 mL of 0.1 M sodium hydroxide (NaOH) in 3.5% sodium chloride (NaCl) using an UltraTurrax homogenizer (IKA Werke GmbH, Staufen, Germany). The homogenates were incubated at 60 °C for 90 min before centrifugation at 4000× *g* for 30 min at 4 °C. The supernatants were frozen and kept at −20 °C until analyses. 

#### 2.2.2. The Good’s Buffer Extraction

This extraction was performed as described by Alhamdani et al. [10] with minor modifications. Raw materials were homogenized 1:6 (*w*:*v*) with a buffer consisting of 20 mM 4-(2-hydroxyethyl)piperazine-1-ethanesulfonic acid (HEPES), 1 mg magnesium chloride (MgCl_2_) and 0.5% 3-((3-holamidopropyl)dimethylammonio)-1-propanesulfonate (CHAPS), pH 7.9 using an UltraTurrax homogenizer. The homogenates were incubated on ice for 30 min with occasional mixing, before centrifugation at 4000× *g*, for 20 min at 4 °C. The supernatants were frozen and kept at −20 °C until analyses. 

### 2.3. Direct Protein Determination

#### Amino Acid Analysis

Sample preparations for analysis of total amino acids were performed as described by Mæhre et al. [11]. For the raw material samples, approximately 200 mg of fish and shrimp samples and approximately 50 mg of flour and dulse samples, were dissolved in 0.7 mL distilled H_2_O and 0.5 mL 20 mM norleucine (internal standard). For the protein extract samples, 500 µL extract was mixed with 50 µL 20 mM norleucine. Subsequently, for all samples, concentrated hydrochloric acid (HCl, 12 M) was added, to a final concentration of 6 M. The sample mixtures were flushed with nitrogen gas for 15 s in order to minimize oxidation, before hydrolysis at 110 °C for 24 h according to Moore and Stein [12]. Following hydrolysis, 100 µL aliquots of the hydrolysates were evaporated under nitrogen gas until complete dryness and re-dissolved to a suitable concentration in lithium citrate buffer at pH 2.2. All amino acids were analyzed chromatographically using an ion exchange column followed by ninhydrin post column derivatization on a Biochrom 30 amino acid analyzer (Biochrom Co., Cambridge, UK). Amino acid residues were identified using the A9906 physiological amino acids standard (Sigma Chemical Co., St. Louis, MO, USA) as described previously [13]. Protein content was calculated as the sum of individual amino acid residues (the molecular weight of each amino acid after subtraction of the molecular weight of H_2_O).

### 2.4. Indirect Protein Determinations

#### 2.4.1. The Kjeldahl Method

The Kjeldahl method was performed according to method 981.10 of the AOAC International [14]. Approximately 1 g of raw material was hydrolyzed with 15 mL concentrated sulfuric acid (H_2_SO_4_) containing two copper catalyst tablets in a heat block (Kjeltec system 2020 digestor, Tecator Inc., Herndon, VA, USA) at 420 °C for 2 h. After cooling, H_2_O was added to the hydrolysates before neutralization and titration. The amount of total nitrogen in the raw materials were multiplied with both the traditional conversion factor of 6.25 [15] and species-specific conversion factors [7,16] in order to determine total protein content. The species-specific conversion factors were 5.6 for fish and shrimp, 5.4 for flours and 4.59 for seaweed, respectively. 

#### 2.4.2. The Modified Lowry Method

The modified Lowry protein measurement was conducted according to the method described by Hartree [17]. The assay was carried out by diluting the extracts to 1 mL with H_2_O and adding 0.9 mL of solution A (2 g L^−1^ potassium sodium tartrate (KNaC_4_H_4_O_6_·4H_2_O) and 100 g L^−1^ sodium carbonate (Na_2_CO_3_) in 0.5 M NaOH) before incubation for 10 min at 50 °C. Following this, the samples were cooled down to room temperature, added 1 mL of solution B (0.2 g L^−1^ KNaC_4_H_4_O_6_·4H_2_O and 0.1 g L^−1^ copper sulfate pentahydrate (CuSO_4_·5H_2_O) in 0.1 M NaOH) and left for 10 min. Finally, 3 mL of solution C (Folin–Ciocalteu phenol reagent in H_2_O (1:16 *v*/*v*)) was added before incubation for 10 min at 50 °C. A standard curve was made of bovine serum albumin (BSA; 0, 0.0625, 0.125, 0.25, 0.5 and 1 g L^−1^) and absorbance was read at 650 nm. 

#### 2.4.3. The Bradford Method

The Bradford assay was conducted according to the method described by Bradford [18]. Briefly, 100 mg Coomassie Brilliant Blue G-250 was dissolved in 50 mL 95% ethanol (C_2_H_5_OH). Thereafter, 100 mL of 85% phosphoric acid (H_3_PO_4_) was carefully added under stirring, before H_2_O was added to a total volume of 1 L. The solution was filtered and kept at 4 °C. For the measurements, 100 µL extract and 5 mL Bradford solution were mixed and incubated for 5 min. A standard curve was made of BSA (0, 0.0625, 0.125, 0.25, 0.5 and 1 g L^−1^) and absorbance was read at 595 nm. 

### 2.5. Statistics Description

All results are presented as arithmetic mean of 5 parallels ± standard deviation (SD). Statistical package for the social sciences 23 (SPSS Inc., Chicago, IL, USA) was used to perform statistical analyses. Shapiro-Wilk’s test for normality and Levene’s test for homogeneity of variance were performed and for samples returning normal distribution, one-way analysis of variance (ANOVA) was performed. For non-normal distributions, the non-parametric Mann-Whitney U test was used. For evaluation of statistics, Tukey and Dunnett’s T3 post-hoc tests were run for equal and un-equal variances, respectively. Means were considered significantly different at *p* < 0.05.

## 3. Results and Discussion

### 3.1. Direct Protein Determination

#### Amino Acid Analysis

Amino acid analysis is one of the analytical principles for protein determination. Here, the proteins are broken down into their constituent amino acids by hydrolysis of the peptide bonds. The liberated amino acid residues are then determined, most often chromatographically, and protein content is calculated as the sum of individual amino acid residues after subtraction of the molecular mass of H_2_O. One major drawback of this method, however, is the protein hydrolysis prior to analysis. The commonest method is hydrolysis in 6 M HCl at 110 °C for 24 h, as described by Moore and Stein [12]. This procedure efficiently hydrolyzes most of the peptide bonds, but at the same time the content of some amino acids are reduced or even destroyed completely [19], and thus, the protein content analyzed by this method may be underestimated. Another drawback of this method is the costs related to investment and analysis. This makes the method unavailable for many food science laboratories. Due to amino acid analysis being the only protein analysis method determining protein contents directly (based solely on amino acid residues and where interfering substances do not affect the results), it was decided to use this method as the reference method in this study and compare all other analyses with the results from this. This is also in accordance with the recommendations given by the Food and Agricultural Organization of the United Nations (FAO) regarding determination of food proteins [20].

### 3.2. Indirect Protein Determination

#### 3.2.1. Protein Content Based on Nitrogen Determination

Some of the most frequently used methods for food protein determination are based on analysis of the total nitrogen content in the samples. Examples of such methods are the Dumas method [21] and the Kjeldahl method [15]. In both methods, the total nitrogen in the sample is liberated at high temperature. In the Kjeldahl method, the nitrogen is released into a strong acid and the content is measured after neutralization and titration. In the Dumas method, the nitrogen is liberated in a gaseous form and is determined with a thermal conductivity detector, after removal of carbon dioxide and water aerosols. The Kjeldahl method was chosen as an example of this analytical principle in this study as it is still recognized as the official method for food protein determination by the AOAC International [14]. Following the nitrogen determination, crude protein content is calculated using a conversion factor. The original, and still frequently used, conversion factor 6.25 is based on an assumption that the general nitrogen content in food proteins is 16% and that all nitrogen in foods is protein-bound. These are, however, quite rough assumptions as the relative nitrogen content varies between amino acids and amino acid composition varies between food proteins [22]. In addition, a wide range of other compounds, such as nitrate, ammonia, urea, nucleic acids, free amino acids, chlorophylls and alkaloids contain nitrogen. These compounds are called non-protein nitrogen and their relative contents are often higher in vegetables than in foods of animal origin [5]. Throughout the years, it has been proven that the conversion factor of 6.25 in most cases overestimates the protein content and in order to adjust for these variations, several species-specific conversion factors have been suggested [6,7,16,22], making the conversion from nitrogen to protein more precise.

#### 3.2.2. Comparison between Amino Acid Analysis and the Kjeldahl Method

In Table 1 the results of the amino acid analysis and the Kjeldahl analysis of the raw materials are shown. Both the traditional conversion factor of 6.25 and the respective species-specific conversion factors are presented. For fish, shrimp and flour, the species-specific factors used in these calculations were the average conversion factors suggested by Mariotti et al. [7], namely 5.6 for fish and shrimp and 5.4 for cereals. The conversion factor used for dulse is the factor suggested by Lourenço et al. [16] as an average for red algae, namely 4.59.

As expected, all of the Kjeldahl results using the traditional conversion factor were significantly higher, ranging from 44% to 71% higher, than the corresponding amino acid analysis results. More surprisingly, and except for the red algae, the species-specific conversion factors also gave significantly higher protein content than the amino acid analysis. One possible explanation may be that the concentration of some of the amino acids in the sample was reduced as a result of the hydrolysis process prior to the amino acid analysis and that the protein concentration calculated from this analysis was in fact underestimated. Another possible explanation could be that the “real” conversion factors for these species should in fact be even lower than the average values reported by Mariotti et al. [7]. Calculating conversion factors based on the results from this study gave factors of 4.9 for fish and shrimp and 4.7 for flours, respectively.

There are risks associated with calculating protein from nitrogen and the resulting overestimation of protein content. One is the possibility of food adulteration, as a high protein content often raises the economic value of a product [23]. There have been some cases where the producer in order to increase the apparent protein content [24], and subsequently the economic value of the food product have added non-protein nitrogen, such as melamine. This may compromise food safety for consumers and hence, it is important that such food adulteration is rendered impossible. Another risk is that the utilization potential and economic feasibility of “new” raw materials could be overestimated, as protein is one of the constituents with value-added potential in a product. Overestimation could thus give false premises for the establishment of new industries. For instance, the interest of increased industrial utilization of seaweeds has increased greatly the last decades. Several studies have presented a protein content of some red seaweed species of 30–45% [25,26], while when analyzed with amino acid analysis, it commonly ranges between 10% and 20% [11,27,28]. Such a difference could be crucial for the economy of small scale industries. 

#### 3.2.3. Spectrophotometric Methods and Protein Extraction

The third common analytical technique for protein analysis is spectrophotometry. Here, the principle is that functional groups or regions within the protein absorb light in the ultraviolet or visible range of the electromagnetic spectrum (200–800 nm). This absorbance is read and compared with known protein standards. Examples of such functional groups or regions are basic groups, aromatic groups, peptide bonds or aggregated proteins. 

While both nitrogen analysis and amino acid analysis may be performed without any pre-treatment of the raw material, extraction of proteins is a prerequisite before submitting the material to spectrophotometric analysis. Also for protein extraction, the available methods are many. Most common protein extraction protocols are based on exposure of tissue to weak buffers or water, leading to collapse of cells with subsequent release of intracellular proteins as a response to the hypotonic shock that arises. This is very efficient for tissues containing cells without cell walls (animal cells), but not as efficient for cells with cell walls (plant cells). The latter being due to the cell walls protecting the cell against collapse [29]. In this study both protein sources of animal origin and protein sources of plant origin were included in order to evaluate these differences. 

Of the commonest protein extraction methods, especially prior to electrophoresis, is the use of so-called Good’s buffers, a range of buffers first described in 1966 by Good et al. [8]. These buffers contain chemicals with zwitterionic properties, along with one or more detergents and are known to be highly compatible to biological analyses. They are highly water soluble, have low salt-effects and minimal interference with biological functions [10]. The main drawback of these protocols is that they involve several expensive chemicals and some of the chemicals are suspected to be health damaging. In this study, a mixture of HEPES (zwitterionic agent) and CHAPS (detergent) was chosen as an example of this principle.

Proteins are often divided into four main classes, based on their solubility properties. The four classes are albumins that are water-soluble, globulins that are soluble in weak ionic solutions, glutelins that are soluble in weak acidic or basic solutions and prolamines that are soluble in 70% ethanol. Most foods are complex matrices, probably holding several of these protein classes and combining several solutes would probably optimize the extraction yield. In Mæhre et al. [9] it was shown that combining H_2_O, 0.1 M NaOH and 3.5% NaCl at elevated temperature increased extraction yield compared to traditional extraction methods and this method was thus chosen as an example of a simpler protein extraction method in this study.

In Table 2, the extraction yields of the two different extraction methods are presented as protein content relative to the respective raw materials, calculated after amino acid analysis. As seen, extracting proteins using the HEPES/CHAPS protocol was quite ineffective, resulting in very low yields for all raw materials. Salt/alkaline extraction was shown to give significantly higher yields for all raw materials. Further, it was shown that the salt/alkaline protocol was very efficient for extracting proteins of animal origin, managing to extract more or less 100% of the protein. It was also quite efficient for extracting proteins from highly processed plant raw material (white flour), giving a yield of around 80%. The extraction yield was, however, lower in less processed and more complex plant materials, such as whole flour and dulse, the latter being comparable to a previous study [9].

One of the differences between the two chosen extraction methods was that salt/alkaline extraction was performed at 60 °C while HEPES/CHAPS was performed on ice and this could contribute to the difference in extraction yield. Extraction on ice probably protects the proteins against degradation, while heat treatment could possibly accelerate this. Thus, the extraction method should be chosen based on the purpose of further use. However, in this study the goal was merely to examine the differences in protein extraction yield between methods, and thus, protein degradation was not analyzed. 

Following extraction, protein determination using two different spectrophotometric methods, the Bradford method [18] and a modified Lowry method [17], was tested and compared with amino acid analysis. In the Bradford method, Coomassie G-250 dye reacts with ionizable groups on the protein disrupting the proteins tertiary structure and exposing the hydrophobic pockets. This is followed by the dye binding to the hydrophobic amino acids forming stable complexes that can be read at 595 nm [3]. The modified Lowry method is a combination of the Biuret method, where copper ions react with the peptide bonds within the protein, and a reaction between Folin-Ciocalteu reagent and the ring structure on aromatic amino acids. The total reaction forms a stable, dark blue complex that can be read at 650–750 nm [3].

#### 3.2.4. Comparison between Amino Acid Analysis and the Spectrophotometric Methods

In Table 3, the results of protein determination by amino acid analysis, Bradford method and modified Lowry method are shown for the salt/alkaline extracts, while in Table 4 the same analyses are shown for the HEPES/CHAPS extracts. In the salt/alkaline extracts, both the Bradford method and the modified Lowry method gave higher protein estimates than the amino acid analysis for proteins of animal origin. The same was observed when using the modified Lowry method for plant proteins, while the Bradford method resulted in equal or lower protein estimates than the amino acid analysis. In the HEPES/CHAPS extracts, it was not possible to obtain any results using the modified Lowry method. The Bradford method gave higher protein estimates than the amino acid analysis for all raw materials except for the dulse. 

The Lowry method has been widely used for protein determination for many decades, due to its simplicity and availability. However, besides aromatic amino acids, a wide range of other compounds react with the Folin–Ciocalteu reagent [30]. In complex food matrices, containing several interfering compounds, this normally leads to an overestimation of the protein content, which is also what the results from the salt/alkaline extraction show. One of the compounds that has been shown to interfere with the Lowry protocol is HEPES [31]. In this study, the reaction between the HEPES/CHAPS buffer and the Lowry reagent produced a very strong background color in both standards and extracts, making it impossible to obtain accurate protein results. Normally, the Bradford method has been recognized to be less prone to such interference. However, the results from this study show that also for several of the raw materials the protein estimates are very high compared to the amino acid analysis, indicating that there could be some kind of interference. Differences in extraction efficiency of different amino acids could also be a possible explanation. In the Bradford analysis, the basic amino acids contribute more to the final color than do other amino acids [3], while aromatic amino acid contribute more to the color development in the modified Lowry method. As seen in Table 5, there were some significant differences in the extraction efficiency between hydrophobic (proline, glycine, alanine, valine, isoleucine, leucine and phenylalanine), aromatic (tyrosine, phenylalanine, tryptophan and histidine) and basic (lysine, histidine and arginine) amino acids. However, this effect varied between species and the only significant effect when looking at all materials was that the HEPES/CHAPS method seemed to extract hydrophobic amino acids more efficiently than the other methods. 

## 4. Conclusions

As shown, there are many methods available for protein determination and all of them have their advantages and disadvantages. This great assortment of methods makes direct comparison between studies difficult and the choice of analytical method should thus be justified depending on the purpose of the study. 

The results from this study show that protein determination based on nitrogen analysis for most food matrices overestimates the protein content compared to amino acid analysis, whether or not the species-specific conversion factors are used. Spectrophotometric protein determination methods are often affected by interfering substances and could thus overestimate the protein content. Protein extraction often involves chemicals affecting both extraction yield and subsequent determination. This makes such protocols very dependent on the choice of buffers used and methods involving extraction should thus not be the primary choice for food purposes. However, if the purpose is further analytical use, such methods could be a suitable alternative. Amino acid analysis is the only protein analysis method where interfering substances do not affect the results. Although there is potential for improvement in regards to the hydrolysis method, we recommend that this method should be the preferred for food protein determination.

## Figures and Tables

**Table 1:** Protein content from amino acid analysis and Kjeldahl nitrogen analysis of cod, salmon, shrimp, white and whole wheat flour and dulse (red seaweed).

Raw Materials			
Species	Amino Acid Analysis	Kjeldahl (Factor 6.25)	Kjeldahl (Species-Specific Factors)
Cod	111.6 ± 9.1 ^a^	185.7 ± 15.0 ^b^	166.4 ± 13.4 ^b^ (5.6)
Salmon	121.4 ± 6.3 ^a^	208.1 ± 7.3 ^c^	186.5 ± 6.6 ^b^ (5.6)
Shrimp	83.8 ± 8.6 ^a^	132.7 ± 4.7 ^c^	117.8 ± 4.4 ^b^ (5.6)
White flour (wheat)	77.9 ± 6.5 ^a^	117.6 ± 7.3 ^c^	101.6 ± 6.3 ^b^ (5.4)
Whole flour (wheat)	88.2 ± 3.8 ^a^	133.4 ± 7.8 ^c^	115.3 ± 6.8 ^b^ (5.4)
Dulse (red seaweed)	105.3 ± 9.1 ^a^	152.1 ± 10.0 ^b^	111.7 ± 7.3 ^a^ (4.59)

The conversion factors used for the Kjeldahl analysis are the commonly used conversion factor 6.25 and species-specific conversion factors (in parentheses) 5.4 for the flours, 5.6 for fish and shrimp [7] and 4.59 for seaweed [15]. Values are reported as mean value ± standard deviation (SD) (*n* = 5) and in g protein kg^−1^ raw material. Different letters within the same row indicate significant differences between analyses within each species.

**Table 2:** Protein extraction yields calculated from amino acid analysis of cod, salmon, shrimp, white and whole wheat flour and dulse.

Raw Materials		
Species	Extraction Yield Salt/Alkaline	Extraction Yield HEPES/CHAPS
Cod	106.8 ± 11.2 ^b^	13.8 ± 3.0 ^a^
Salmon	99.9 ± 13.3 ^b^	31.9 ± 2.1 ^a^
Shrimp	113.3 ± 17.0 ^b^	14.4 ± 2.2 ^a^
White wheat flour	78.5 ± 11.1 ^b^	15.0 ± 3.7 ^a^
Whole wheat flour	66.1 ± 20.9 ^b^	20.4 ± 3.2 ^a^
Dulse (red seaweed)	34.9 ± 6.7 ^b^	12.0 ± 1.6 ^a^

Values are reported as mean value ± SD (*n* = 5) and in % of amino acid content in the respective raw materials (as shown in Table 1). Different letters within the same row indicate significant differences between the extraction yields of the different methods within each species. HEPES: 4-(2-hydroxyethyl)piperazine-1-ethanesulfonic acid; CHAPS: 3-((3-holamidopropyl)dimethylammonio)-1-propanesulfonate.

**Table 3:** Protein content calculated from amino acid analysis, Bradford analysis and modified Lowry analysis in salt/alkaline protein extracts of cod, salmon, shrimp, white and whole wheat flour and dulse.

Salt/Alkaline Protein Extracts			
Species	Amino Acid Analysis	Bradford	Modified Lowry
Cod	118.8 ± 9.8 ^a^	198.7 ± 24.1 ^b^	194.5 ± 11.4 ^b^
Salmon	120.9 ± 13.9 ^a^	234.6 ± 10.6 ^c^	211.9 ± 9.2 ^b^
Shrimp	93.9 ± 7.8 ^a^	165.5 ± 10.3 ^b^	151.2 ± 8.2 ^b^
White flour (wheat)	60.9 ± 8.1 ^b^	45.0 ± 6.6 ^a^	85.7 ± 12.3 ^c^
Whole flour (wheat)	58.2 ± 18.6 ^a^	46.2 ± 18.5 ^a^	88.8 ± 27.3 ^b^
Dulse (red seaweed)	36.9 ± 9.1 ^a^	48.4 ± 3.9 ^a^	89.5 ± 15.9 ^b^

Values are reported as mean value ± SD (*n* = 5) and in g protein kg^−1^ raw material. Different letters within the same row indicate significant differences between methods within each species.

**Table 4:** Protein content calculated from amino acid analysis, Bradford analysis and modified Lowry analysis in HEPES/CHAPS protein extracts of cod, salmon, shrimp, white and whole wheat flour and dulse.

HEPES/CHAPS Protein Extracts			
Species	Amino Acid Analysis	Bradford	Modified Lowry
Cod	15.3 ± 2.7 ^a^	39.4 ± 7.5 ^b^	n.a.
Salmon	38.7 ± 3.3 ^a^	98.4 ± 3.1 ^b^	n.a.
Shrimp	11.9 ± 0.7 ^a^	25.7 ± 1.6 ^b^	n.a.
White flour (wheat)	11.6 ± 2.7 ^a^	18.5 ± 3.9 ^b^	n.a.
Whole flour (wheat)	18.0 ± 2.8 ^a^	30.0 ± 5.0 ^b^	n.a.
Dulse (red seaweed)	12.5 ± 1.4 ^b^	7.8 ± 1.3 ^a^	n.a.

Values are reported as mean value ± SD (*n* = 5) and in g protein kg^−1^ raw material. Different letters within the same row indicate significant differences between methods within each species. n.a.: not applicable.

**Table 5:** Relative amount of hydrophobic, aromatic and basic amino acids (AA) in raw materials and protein extracts after extraction using HEPES/CHAPS and salt/alkaline in cod, salmon, shrimp, white wheat flour, whole wheat flour and dulse, as well as across all species.

		Raw Material	HEPES/CHAPS	Salt/Alkaline
Cod	Hydrophobic AA	37.8 ± 0.9 ^ab^	42.8 ± 3.2 ^b^	35.7 ± 1.3 ^a^
	Aromatic AA	9.3 ± 1.7	9.4 ± 2.4	8.0 ± 2.7
	Basic AA	19.6 ± 0.3	19.2 ± 1.2	18.7 ± 0.6
Salmon	Hydrophobic AA	39.4 ± 1.0 ^a^	44.3 ± 1.0 ^b^	39.5 ± 1.6 ^a^
	Aromatic AA	10.1 ± 1.4	9.0 ± 3.3	8.7 ± 3.0
	Basic AA	20.0 ± 0.2	18.2 ± 1.4	20.0 ± 3.1
Shrimp	Hydrophobic AA	38.4 ± 0.4 ^a^	47.3 ± 1.2 ^b^	38.0 ± 2.5 ^a^
	Aromatic AA	10.2 ± 0.9 ^b^	4.2 ± 0.9 ^a^	8.7 ± 2.1 ^b^
	Basic AA	19.8 ± 0.2 ^b^	17.6 ± 1.1 ^a^	19.8 ± 0.7 ^b^
White flour (wheat)	Hydrophobic AA	41.2 ± 0.9 ^b^	40.5 ± 1.4 ^b^	37.4 ± 0.6 ^a^
	Aromatic AA	8.9 ± 1.1 ^a^	9.4 ± 2.7 ^ab^	11.5 ± 0.5 ^b^
	Basic AA	8.6 ± 0.3 ^a^	11.0 ± 0.5 ^b^	7.9 ± 0.7 ^a^
Whole flour (wheat)	Hydrophobic AA	40.9 ± 0.5 ^b^	38.6 ± 0.6 ^a^	40.0 ± 1.7 ^ab^
	Aromatic AA	9.4 ± 1.1	10.6 ± 0.3	8.1 ± 2.2
	Basic AA	10.2 ± 0.2 ^b^	12.4 ± 0.5 ^c^	8.4 ± 0.4 ^a^
Dulse (red seaweed)	Hydrophobic AA	43.5 ± 1.4 ^b^	34.6 ± 2.7 ^a^	46.3 ± 2.3 ^b^
	Aromatic AA	10.3 ± 0.5 ^b^	1.6 ± 0.2 ^a^	9.3 ± 1.8 ^b^
	Basic AA	14.0 ± 1.7 ^c^	4.4 ± 0.8 ^a^	6.9 ± 0.7 ^b^
All species	Hydrophobic AA	40.2 ± 2.1 ^ab^	41.4 ± 4.5 ^b^	39.5 ± 3.8 ^a^
	Aromatic AA	9.7 ± 1.2	7.4 ± 3.8	9.1 ± 2.3
	Basic AA	15.4 ± 4.8	13.8 ± 5.4	13.6 ± 6.2

Values are given as mean ± SD (*n* = 5) and in % of total amino acids. Different letters within the same row indicate significant difference (*p* < 0.05) between amino acid compositions in raw materials and in the two extracts.
